# Effects of extended abstinence on cognitive functions in tramadol‐dependent patients: A cohort study

**DOI:** 10.1002/npr2.12188

**Published:** 2021-06-15

**Authors:** Shehab H. Hassaan, Hossam Khalifa, Alaa M. Darwish

**Affiliations:** ^1^ Department of Psychiatry Sulaiman Alrajhi University Al Bukayriyah Saudi Arabia; ^2^ Department of Psychiatry Assiut University Assiut Egypt

**Keywords:** abstinence, cognition, trail‐making test, tramadol, Wechsler Adult Intelligence Scale, Wechsler Memory Scale‐Revised

## Abstract

**Background:**

Some pieces of the literature report impaired cognitive functioning in tramadol dependence. Whether extended abstinence improves cognitive functioning or not is not well studied.

**Aim:**

We aimed to measure the change in cognitive functioning following complete abstinence among individuals with tramadol dependence.

**Methods:**

Eighty‐three male tramadol‐dependent (TD) and 57 matched healthy controls participated in this study. Cognitive functions were assessed using: The Trail making test (TMT), Wechsler Memory Scale‐Revised (WMS‐R), and Wechsler Adult Intelligence Scale (WAIS). Patients were assessed in the first week immediately after the end of the in‐patient treatment program (T1), and after six months of sustained abstinence (T2).

**Results:**

At T1, the TD group showed deficits on all tested cognitive parameters (visual attention, task switching, working memory, visual memory, verbal memory, verbal knowledge, Verbal IQ, Performance IQ, and Full‐Scale IQ) in comparison to the control group. At T2, significant improvements had occurred in all the tested parameters except performance IQ. The cognitive performance of the abstinent individuals at T2 was comparable to the control group for the verbal subsets of WMS‐R, Verbal IQ, Performance IQ, and Full‐Scale IQ. Nevertheless, it was still worse than the control group in TMT, and all other WMS subsets.

**Conclusion:**

tramadol dependence has negative effects on cognitive performance, which improves with extended abstinence.

## Abstract 2

This research aimed to measure the change in the cognitive functioning following complete abstinence among individuals with tramadol dependence. The results showed that tramadol dependence has negative effects on cognitive performance, which improves with extended abstinence.

## Graphical abstract

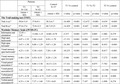

## 1 INTRODUCTION

Tramadol is a synthetic opioid analgesic first introduced in 1977 by a German pharmaceutical company as a pain killer through acting on µ‐opioid receptors by its R‐stereoisomer and S‐stereoisomer.^1^ As an opioid analgesic, it is generally used for management of moderate to severe pain and has likewise been used in the management of sexual dysfunction, such as premature ejaculation.^2^ As a prescription opioid analgesic, it was widely abused in many countries in Asia and Africa, and several studies reported the growing nonmedical use of tramadol in some countries in Africa and Middle East.^3^ World Health Organization (WHO) in 2014 estimated the prevalence of abuse of tramadol in China to be about 14%, while estimated its rate of abuse in the USA to be about 3/100 000.^4^ Egypt encounters an increasing health problem regarding Tramadol dependence (TD) in the last decade. An Egyptian study estimated the prevalence of tramadol abuse in the Egyptian youth to be about 9%.^5^ Another study estimated its abuse to be 48% of all drug abuses in Egypt.^6^

Deficits in different cognitive domains; such as attention/concentration, memory, and psychomotor speed and performance, and also in the processing of emotional stimuli and in executive functions, were extensively studied in drug‐dependent individuals compared with healthy controls.^7^, ^8^, ^9^ Only a few studies have dealt with cognitive deficits in previously opiate (heroin)‐dependent, long‐term abstinent individuals.^10^, ^11^, ^12^

Tramadol, as an opioid analgesic, may result in mild cognitive deficits in attention, complex working memory, and episodic memory when used for a long duration.^13^, ^14^ Some studies found a general intellectual deficit in patients with early or ongoing chronic opioid abuse.^15^, ^16^ On the other hand, there is more consistent evidence for deficits in attention, memory, and executive function.^17^, ^18^, ^19^ Several studies showed that the cognitive functions of the patients during abstinence from opioid abuse is better than cognitive function during opioid abuse.^10^, ^20^, ^21^, ^22^, ^23^ Furthermore, late and early abstinence differ in many aspects regarding the cognitive functions and these differences need more clarification. The first weeks of abstinence are critical for opioid‐dependent patients because most of the relapses and the drop‐outs from the out‐patient programs occur during this period.^24^, ^25^ Therefore, the assessment of cognitive function within this time is relevant.

So far our knowledge goes, much attention was paid for the disturbance of cognitive functions related to the abuse of other substances such as opium, cocaine, heroin, cannabis, methamphetamine, etc But the aforementioned discussion brings about the fact that there is a knowledge gap of the cognitive function after abstinence. We, therefore, wanted to measure the effect of tramadol drug on cognitive functions and whether these cognitive impairments are permanent or they are subject to improve with extended abstinence.

## 2 METHODS

This prospective study assessed the cognitive function after abstinence from tramadol in TD patients compared with healthy controls. The Ethics Committee of Assiut University accepted the study protocol. The study was conducted between January to October 2018. We wanted to assess the cognitive function to reject the null hypothesis that TD patients who were abstinent for a good duration of time would have no change of IQ, memory, attention and executive function when compared to their performance directly after the detoxification program, or compared to the healthy controls.

All participants with TD, met the DSM‐IV criteria for opioid dependence, were volunteers between 18 and 45 years from a series of consecutive patients admitted to Assiut University addiction unit for detoxification and management of drug withdrawal symptoms. Patients with mixed substance abuse, or diagnosed with acute axis I psychiatric comorbidity, according to the Diagnostic and Statistical Manual of Mental Disorders (DSM‐IV) not related to substance, or those who were receiving any psychotropics, especially those which might affect cognition, were excluded. Also, patients with severe brain injury, chronic neurological disease, with a history of epileptic seizures, or primary organic cognitive deficit were excluded from the study. All participants signed informed consent which included information about the nature and purpose of the research; the expected duration of the subject's participation; a statement that participation in research is voluntary; probable benefits associated with research participation; information about data protection/confidentiality/privacy; reference contacts for any further answers to pertinent questions about the research and the subject's rights and a statement offering the subject the opportunity to withdraw at any time from the research and the withdrawal would not affect any management privileges.

A control group was recruited from the patients' relatives or the staff of the same institution. They were matched for age, gender, educational level, and other demographic variables as far as possible. None of the controls had any history of illegal or opioid drug abuse. The controls were screened by psychiatric interview for having no history of significant psychiatric morbidity or substance abuse. The dependence and other psychiatric diagnoses were done according to the Structured Clinical Interview (SCID) for DSM‐IV axes I and II.^26^, ^27^

### 2.1 Cognitive tests

Cognitive performance was determined by a small battery designed to probe different aspects of executive function, memory, and intelligence. All tests were administered manually using paper and pencil testing. The testing battery included:

#### 2.1.1 Trail‐making test part A and B

Part A test is used to measure cognitive and perceptual speed and takes almost 1 minute to complete. The test requires immediate recognition of the symbolic importance of numbers and letters and the flexibility to put them into a sequence under time pressure. Part A measures information processing and psychomotor speed. Part B of this test evaluates cognitive flexibility and the ability to switch between amounts and is 2 minutes long. The patient has to connect numbers and letters in sequential order alternately (eg, A‐1‐B‐2‐C‐3). The score on each part represents the amount of time required to complete the task.^28^ The lesser the score is, the better is the performance.

#### 2.1.2 Wechsler Adult Intelligence Scale (WAIS)

For a broad assessment of general cognitive and intellectual abilities.^29^ The study used Arabic validated version of the WAIS with Egyptian norms as a reference.^30^

#### 2.1.3 Wechsler Memory Scale‐Revised (WMS‐R)

One of the most widely used tests for evaluating memory functions in adults, the scores reflect general, verbal and visual memory, attention/concentration and delayed recall.^31^

### 2.2 Procedure

A total of 136 patients with TD disorder were assessed. Thirteen were excluded for having comorbid substance misuse, and six were excluded due to having comorbid psychiatric diagnosis. Eighty‐three patients completed the study and 34 patients either did not appear during the follow‐up or were tested positive in the urine screen test during the follow‐up visits.

The assessment of each subject took 2‐3 hours. Therefore, the interaction was made a two‐session‐interview. The first session included the clinical and demographic data collection, administration of SCID‐I. The second session was for the administration of cognitive tests. Trained psychiatrists and psychologists, with proper working experience, conducted the interview using the tools. Initial screening (T1) was done in the first week immediately after the end of the in‐patient treatment program. The second assessment (T2) was performed after 6 months of complete abstinence. All subjects received a physical examination, routine laboratory testing while they were in the hospital. Subjects in the drug‐dependent group underwent repeated random urine drug‐screening examinations with the average of 4‐6 screenings for each patient during the 6‐month follow‐up period. Negative urine drug‐screening test results are a condition of participation in this study. The nondrug‐dependent group also underwent the same test.

### 2.3 Statistical analysis

Data were recorded and analyzed using the statistical package of social sciences IBM‐SPSS (version 22).^32^ The quantitative variables were presented as mean ($\bar{x}$) and standard deviation (SD), while qualitative variables were presented as frequency and percentage. We used *χ* ^2^ test for the comparison of categorical variables, while we used independent‐samples *t* test for comparison of continuous variables. Spearman Correlation Test (*r*) was used for assessing the relationship (direction and power) between quantitative variables. A *P*‐value of ≤.05 was set to be statistically significant.

## 3 RESULTS

One hundred forty subjects were enrolled in this study, 83 patients with tramadol dependence and 57 healthy controls. The mean age for the tramadol‐dependence group (27.02 ± 5.70 years) was almost equal to that of (25.7 ± 3.88 years) the nondependence group (*P* = .28). Similarly, the mean educational years was 11.53 ± 3.27 years, compared to 12.44 ± 3.43 years (*P* = .12) in the control group. No statistical difference between patients and controls was found in any demographic variables except marital status (*χ* ^2^ = 30.32, *P* < .001) and occupational status (*χ* ^2^ = 12.32, *P* = .006). TD group were more likely to be single, manual workers and unemployed, compared to controls who were more likely to be married, manual workers and office workers, as shown in Table 1.

**TABLE 1:** Demographic characteristics of patients and controls

	Group				*P* value
	Cases n = 83		Control n = 57		
	No.	%	No.	%	
Age					
18‐29 y old	51	63.3	30	53.3	*χ*^2^ = 1.0767 *df* = 1 .299
30‐45 y old	32	36.7	27	46.7	
Mean ± SD	27.027 ± 5.700		25.777 ± 3.875		*t* = 1.081 .283
Marital status					
Single	55	66.7	10	16.7	*χ*^2^ = 30.321 *df* = 1 <.001**
Married	28	33.3	47	83.3	
Residence					
Urban	36	43.3	23	40	*χ*^2^ = 0.1266 *df* = 1 .7219
Rural	47	56.7	34	60	
Occupation					
Unemployed	25	30.0	13	23.3	*χ*^2^ = 12.318 *df* = 3 .0063*
Student	3	3.3	1	1.7	
Manual work	47	56.7	24	42.3	
Office work	8	10.0	19	33.3	
Socioeconomic level					
High (>145.69)	6	6.7	10	17.5	*χ*^2^ = 3.771 *df* = 2 .1517
Average (75.93‐145.69)	63	76.6	40	70	
Low (<75.93)	14	16.7	7	12.5	
Education					
Illiterate	8	10.0	6	10.5	*χ*^2^ = 4.427 *df* = 3 .2189
Intermediate school	28	33.3	25	43.3	
High school	36	43.3	15	26.3	
University	11	13.3	11	19.3	
Years of education Mean ± SD	11.527 ± 3.267		12.437 ± 3.434		*t* = −1.586 .115

* *P* < .05.

** *P* < .01.

### 3.1 Assessment of general intellectual abilities

While assessing general intellectual abilities with WAIS scale (Table 2), IQ scores in T1 was lower in all of verbal, (*P* = .07), performance (*P* = .002) and total (*P* = .03) in the patient group compared to the control group, though the verbal IQ was just not significant. At T2, the results showed improvement in verbal, performance and the total, mean IQ scores in comparison to T1 scores (*P* = .03, .07 and .02) respectively, though the performance IQ improvement was just not significant. Additionally, T2 IQ scores were homogeneous to those of the control group (*P* = .72 for verbal; .21 for performance; and .94 for total respectively).

**TABLE 2:** Differences in general intellectual abilities between patients with tramadol‐dependence and healthy controls

	At the base line T1 (n = 136)	At 6‐mo follow‐up T2 (n = 83)	Control (n = 57)	T1 vs Control		T1 vs T2		T2 vs Control	
				*t*‐value	*P*‐value	*t*‐value	*P*‐value	*t*‐value	*P*‐value
Verbal WAIS‐R Mean ± SD	83.55 ± 8.9	86.5 ± 6.58	86.1 ± 6.53	−1.844	.067	−2.186	.030*	0.362	.718
Performance WAIS‐R Mean ± SD	85.3 ± 9.4	88.23 ± 9.9	90.53 ± 9.964	−3.157	.002**	−1.807	.073	1.257	.211
Total WAIS‐R Mean ± SD	82.3 ± 8.9	85.62 ± 8.05	85.5 ± 8.2	−2.157	.032*	−2.302	.022*	0.080	.936

Note

T1 Patients' test results in the first week immediately after the end of the in‐patient treatment program. T2 Patients' test results after 6 mo of sustained abstinence.

* *P* < .05.

** *P* < .01.

### 3.2 Assessment of memory functions

Evaluating memory functions by Wechsler Memory scale (WMS‐R) revealed that at T1 assessment, the control group had better performance on tests of memory functions than patients with tramadol dependence in all assessed subtests. Table 3 shows that the differential performance was observed in memory subtests (*P* < .001). At T2, the results showed statistically significant improvement in all assessed subsets except visual paired association I and II (*P* = .08 and .20) respectively. Nevertheless, at T2 the performance was still worse than that of the control group in all WMS subsets except information and orientation, verbal paired association I and II (*P* = .28, .89 and .27 respectively).

**TABLE 3:** Differences in executive and memory functions between patients with tramadol‐dependence and healthy controls

	Patients		Control N = 57 (mean ± SD)	T1 vs control		T1 vs T2		T2 vs control	
	T1 N = 136 (mean ± SD)	T2 N = 83 (mean ± SD)		*t*‐value	*P*‐value	*t*‐value	*P*‐value	*t*‐value	*P*‐value
The Trail‐making test (TMT)									
Trail A (s)^a^	58.8 ± 11.8	37.0 ± 9.1	30.3 ± 6.7	−16.489	<.001	−12.672	<.001	−4.639	<.001
Trail B (s)^a^	97.4 ± 17.6	73.9 ± 12.3	60.7 ± 9.23	−14.429	<.001	−9.447	<.001	−6.558	<.001
Wechsler Memory Scale (WMS‐IV)									
Information and orientation^b^	12.83 ± 2.41	13.80 ± 1.23	14.00 ± 0.78	−3.537	<.001	2.875	.005	−1.086	.279
Digit span backwards^b^	4.07 ± 1.55	5.80 ± 0.41	8.93 ± 1.79	−17.151	<.001	8.494	<.001	−15.377	<.001
Digit span forwards^b^	5.67 ± 1.58	8.00 ± 1.29	9.87 ± 1.28	−16.301	<.001	9.434	<.001	−8.453	<.001
Visual memory span backwards^b^	4.23 ± 1.81	5.30 ± 0.81	7.20 ± 1.49	−10.253	<.001	4.839	<.001	−8.817	<.001
Visual memory span forwards^b^	6.40 ± 2.04	6.93 ± 0.55	8.20 ± 1.24	−5.946	<.001	1.975	.050	−7.272	<.001
Visual paired association I^b^	6.70 ± 3.10	8.00 ± 5.50	14.13 ± 2.51	−17.161	<.001	1.789	.075	−7.702	<.001
Visual paired association II^b^	3.23 ± 1.07	3.50 ± 1.46	5.93 ± 0.25	−18.684	<.001	1.281	.202	−12.394	<.001
Verbal paired association I^b^	9.57 ± 4.78	15.60 ± 4.75	15.07 ± 2.84	−7.789	<.001	7.500	<.001	−0.138	.890
Verbal paired association II^b^	4.33 ± 1.54	7.27 ± 1.68	7.53 ± 0.63	−14.845	<.001	7.187	<.001	−1.098	.274

Note

T1 Patients' test results in the first week immediately after the end of the in‐patient treatment program. T2 Patients' test results after 6 mo of sustained abstinence.

^a^ Higher scores indicate slower function.

^b^ Higher scores indicate better function.

### 3.3 Relation with clinical variables

Most of the clinical variables were significantly correlated with memory functions (Table 4) except the age. The duration of dependence was negatively correlated with memory functions (*P* < .001, digit span backwards *P* < .001, digit span forwards *P* = .03, visual memory span backwards *P* = .001, visual memory span forwards *P* = .01, visual paired association II *P* = .003, and verbal paired association II *P* = .02). Years of education was positively correlated with memory functions (information and orientation *P* < .001, digit span backwards *P* < .001, digit span forwards *P* = .02, visual memory span backwards *P* = .01, visual memory span forwards *P* < .001, visual paired association I *P* < .001, visual paired association II *P* < .001, and verbal paired association I *P* < .001). Socioeconomic status correlated positively with memory functions (Information and orientation *P* = .001, digit span backwards *P* = .003, digit span forwards *P* = .007, visual memory span backwards *P* = .004, verbal paired association, I *P* = .033, and visual paired association II *P* = .007, verbal paired association I *P* = .013, verbal paired association II *P* = .003).

**TABLE 4:** Correlation of clinical variables with cognitive functions in patients with tramadol‐dependence

	Age		Duration of dependence		Socioeconomic status		Years of education	
	*r*	*P* value	*r*	*P* value	*r*	*P* value	*r*	*P* value
Trail A^a^	.021	.590	.**079** *	.**036**	**−.092** *	.**015**	**−.132** **	**<.001**
Trail B^a^	.051	.174	.**085** *	.**026**	**−.075** *	.**050**	**−.126** **	.**001**
WMS‐R								
Information and orientation^b^	−.043	.259	**−.154** **	**<.001**	.**125** **	.**001**	.**203** **	**<.001**
Digit span backwards^b^	**−.170** **	**<.001**	**−.165** **	**<.001**	.**113** **	.**003**	.**219** **	**<.001**
Digit span forwards^b^	−.062	.100	**−.085** *	.**026**	.**102** **	.007	.**085** *	.**023**
Visual memory span backwards^b^	−.032	.393	**−.130** **	.**001**	.**110** **	.**004**	.**098** **	.**010**
Visual memory span forwards^b^	.023	.548	**−.093** *	.**014**	.012	.748	.**147** **	**<.001**
Visual paired association I^b^	−.022	.561	.062	.100	.080*	.033	.**138** **	**<.001**
Visual paired association II^b^	−.025	.506	**−.110** **	.**003**	.**101** **	.**007**	.**165** **	**<.001**
Verbal paired association I^b^	−.019	.612	**−.121** **	.**001**	.**094** *	.**013**	.**175** **	**<.001**
Verbal paired association II^b^	−.073	.055	**−.089** *	.**017**	.**111** **	.**003**	.**169** **	**<.001**
Total IQ	.004	.914	**−.112**	.**003**	.024	.518	.**074** *	.**050**

Bold values are those with significant *P*‐value.

* *P* < .05.

** *P* < .01.

^a^ Higher scores indicate better function.

^b^ Higher scores indicate slower function.

### 3.4 Assessment of attention and executive functions

On assessment with trail‐making test (TMT) A and B (Table 3), statistically significant differences were observed between the study and control groups (*P* < .001 in both). The study group performed poorly compared with the control group at T1. At T2, there was significant improvement in both tests A (*P = *<.001) and B (*P = *<.001). Although the patients' performance in TMT improved in comparison to their performance in the first assessment (T1), it is still worse than the performance of the unexposed group (*P* = <.001; *P* = <.001).

Trail‐making test (Table 4), was positively correlated with duration of dependence (test A *P* = .04, test B *P* = .03). Socioeconomic status was negatively correlated with the TMT mean scores (Test A *P* = .02, test B *P* = .02). Additionally, years of education was negatively correlated with the TMT mean scores (Test A *P* < .001, test B *P* = .001).

## 4 DISCUSSION

Our study aimed to explore the cognitive function of extended abstinence from tramadol in TD patients. We expected that this study would throw the light on the effect of extended abstinence on cognitive function in TD patients in line with our hypothesis.

On the first assessment (T1), the patients showed cognitive deficits on all tested cognitive parameters (visual attention, task switching, working memory, visual memory, verbal memory, verbal knowledge, Verbal IQ, Performance IQ, and Full‐Scale IQ) in comparison to the control group. However, after 6 months of abstinence, there was a marked improvement in the cognitive functions of tramadol‐dependent patients in comparison to their previous performance directly after discharge from the detoxification program. Unsurprisingly, the cognitive functions of those patients, even after 6‐month of abstinence, are still mildly below the level of the control group.

We studied the correlation of different clinical variables with cognitive functions. We found that increase years of education and higher socioeconomic status were associated with better performance in the cognitive tests. On the contrary, the duration of tramadol dependence is associated with lower performance in cognitive testing.

Our results concur with previous studies that investigated the effect of other opioids on cognitive performance. Prosser et al^33^ found deterioration in visual perception and memory, intelligence and attention among other cognitive domains in patients on methadone maintenance. The results are also consistent with the findings of Vella Brincat and Macleod who studied the psychomotor impairment in opioid dependence.^34^

Several pieces of literature explained for cognitive deficits during early opioid abstinence. Several neural dysregulations events occur during that time. Examples of these dysregulations are changes in the striatum and the limbic system related to the downregulation of mu opiate receptors activity associated with elevation of gamma‐aminobutyric acid (GABA) and dynorphin release.^35^ These changes are followed by noradrenaline increased in the locus coerulus which is accompanied by excessive glutamate in the hippocampus and the anterior cingulate cortex.^36^ Moreover, noradrenergic activation of corticotrophin‐releasing factor and the resulting brain stress systems hyperactivity, plays an essential role in cognitive dysfunction exhibited by those patients.^37^ It has been postulated that both episodic and working memory are impaired with chronic stress system hyperactivity.^38^, ^39^ The results of Mintzer et al study are in partial agreement with our results. In their study, they found a working memory deficit in opioid‐dependent patients on methadone maintenance, while patients with 9 months of opioid abstinence showed normal performance.^11^ We found a marked improvement in cognitive functions after 6 months of abstinence but not to the level of healthy controls. These differences could be explained by the difference in duration of abstinence, which is longer in Mintzer's study.

Some studies indicated that executive function deficit may be found during late opioid abstinence as well^22^, ^23^ which agreed with our results that showed a mild cognitive deficit in TMT in comparison to the control group even after 6 months of abstinence. On the contrary to the effect of single substance abuse on cognitive functions, polysubstance‐dependent individuals usually suffer long‐lasting neuropsychological impairment with a mild recovery of functioning.^40^

The negative correlations that were found between memory functions, intelligence tests, executive functions and duration of dependence are in agreement with the previously mentioned neural dysregulations and the increase of its severity with prolonged substance dependence. On the other hand, the positive correlation that was found between cognitive functions and the years of education was in agreement with Latvala et al^41^ who reported in their population‐based study that Poorer verbal intellectual ability was accounted for by parental and own low basic education.

## 5 CLINICAL IMPLICATIONS

During the early stage of abstinence, the higher cognitive functions are essential to consolidate recovery and to take control over the impulsive‐compulsive brain circuits. Hence, identifying cognitive deficits could be a useful tool in tailoring more efficient management plans. Additionally, the results of this study highlight the benefits of cognitive behavioral therapy in improving recovery outcomes.

## 6 CONCLUSION

Our results showed that Tramadol dependence has deleterious effects on cognitive performance, which improves rapidly when abstinence is extended. The improvement in cognitive functions was not complete, and the performance in cognitive tests was still below the level of nondependent controls. More years of education and higher socioeconomic status were associated with better cognitive functions. On the contrary, longer duration of dependence was associated with worse cognitive functions.

## 7 STRENGTHS AND LIMITATIONS

As far as we know, this is the first Egyptian study addressing the issue of cognitive functions in TD patients in a prospective way. The study also used specific and validated psychometric tools for the assessment of multiple cognitive functions. The relatively small sample size, the long duration of follow‐up visits and the increased number of drop‐outs were some major limitations of our study.

## CONFLICT OF INTEREST

All authors declare no actual or potential conflict of interest whether financial, personal or otherwise related to this manuscript.

## AUTHOR CONTRIBUTIONS

Shehab Hassaan: study concept and design, patients' interview, gathering and analysis of the data, drafting and critical review of manuscript; Hossam Khalifa: study design, patients' interview, gathering and analysis of the results and writing the main manuscript; Alaa Darwish: analysis of the results and review of the manuscript. All authors approved the final manuscript.

## ETHICAL APPROVAL

“All procedures followed were in accordance with the ethical standards of the responsible committee on human experimentation (institutional and national) and with the Helsinki Declaration of 1975, as revised in 2000 (5). The Ethics Committee of Assiut University accepted the study protocol and Informed consents were obtained from all patients for being included in the study.” Approval of the research protocol by an Institutional Reviewer Board: Approved from The Ethics Committee of Assiut University.

## INFORMED CONSENT

Written informed consent were obtained from all patients for being included in the study.

## Data Availability

The data are not publicly available due to privacy and ethical restrictions. The raw data belonged to the present study cannot be made publicly available, because the disclosure of personal data was not included in the research protocol of the present study.

## References

[1] Kupper RJ, Stumpf A. Synthesis of (+/‐)‐2‐((dimethylamino) methyl)‐1‐(aryl) cyclohexanols. In: Google Patents. 2003.

[2] Salem EA, Wilson SK, Bissada NK, Delk JR, Hellstrom WJ, Cleves MA. Tramadol HCL has promise in on‐demand use to treat premature ejaculation. J Sex Med. 2008;5(1):188–93. 17362279 10.1111/j.1743-6109.2006.00424.x

[3] Gowing LR, Ali RL, Allsop S, Marsden J, Turf EE, West R, et al. Global statistics on addictive behaviours: 2014 status report. Addiction. 2015;110(6):904–19. 25963869 10.1111/add.12899

[4] Organization WH. Global status report on road safety 2015. Geneva: World Health Organization; 2015.

[5] Bassiony MM, Salah El‐Deen GM, Yousef U, Raya Y, Abdel‐Ghani MM, El‐Gohari H, et al. Adolescent tramadol use and abuse in Egypt. Am J Drug Alcohol Abuse. 2015;41(3):206–11. 25859610 10.3109/00952990.2015.1014959

[6] Mohamed N, El Hamrawy L, Shalaby A, El Bahy M, Abd Allah M. An epidemiological study of tramadol HCl dependence in an outpatient addiction clinic at Heliopolis Psychiatric Hospital. Menoufia Med J. 2015;28(2):591–6.

[7] Specka M, Finkbeiner T, Lodemann E, Leifert K, Kluwig J, Gastpar M. Cognitive‐motor performance of methadone‐maintained patients. Eur Addict Res. 2000;6(1):8–19. 10729738 10.1159/000019004

[8] Strand MC, Fjeld B, Arnestad M, Mørland J. Can patients receiving opioid maintenance therapy safely drive? A systematic review of epidemiological and experimental studies on driving ability with a focus on concomitant methadone or buprenorphine administration. Traffic Inj Prev. 2013;14(1):26–38. 23259516 10.1080/15389588.2012.689451

[9] Verdejo A, Toribio I, Orozco C, Puente KL, Pérez‐García M. Neuropsychological functioning in methadone maintenance patients versus abstinent heroin abusers. Drug Alcohol Depend. 2005;78(3):283–8. 15893159 10.1016/j.drugalcdep.2004.11.006

[10] Davis PE, Liddiard H, McMillan TM. Neuropsychological deficits and opiate abuse. Drug Alcohol Depend. 2002;67(1):105–8. 12062785 10.1016/s0376-8716(02)00012-1

[11] Mintzer MZ, Copersino ML, Stitzer ML. Opioid abuse and cognitive performance. Drug Alcohol Depend. 2005;78(2):225–30. 15845327 10.1016/j.drugalcdep.2004.10.008

[12] Verdejo‐García A, Pérez‐García M. Profile of executive deficits in cocaine and heroin polysubstance users: common and differential effects on separate executive components. Psychopharmacology. 2007;190(4):517–30. 17136401 10.1007/s00213-006-0632-8

[13] Kamboj SK, Tookman A, Jones L, Curran HV. The effects of immediate‐release morphine on cognitive functioning in patients receiving chronic opioid therapy in palliative care. Pain. 2005;117(3):388–95. 16198201 10.1016/j.pain.2005.06.022

[14] Sjøgren P, Christrup LL, Petersen MA, Højsted J. Neuropsychological assessment of chronic non‐malignant pain patients treated in a multidisciplinary pain centre. Eur J Pain. 2005;9(4):453–62. 15979026 10.1016/j.ejpain.2004.10.005

[15] Grant I, Adams KM, Carlin AS, Rennick PM, Judd LL, Schooff K. The collaborative neuropsychological study of polydrug users. Arch Gen Psychiatry. 1978;35(9):1063–74.

[16] Rounsaville BJ, Jones C, Novelly RA, Kleber H. Neuropsychological functioning in opiate addicts. J Nerv Ment Dis. 1982;170(4):209–16. 7062007 10.1097/00005053-198204000-00005

[17] Ornstein TJ, Iddon JL, Baldacchino AM, Sahakian BJ, London M, Everitt BJ, et al. Profiles of cognitive dysfunction in chronic amphetamine and heroin abusers. Neuropsychopharmacology. 2000;23(2):113–26. 10882838 10.1016/S0893-133X(00)00097-X

[18] Rogers RD, Everitt BJ, Baldacchino A, Blackshaw AJ, Swainson R, Wynne K, et al. Dissociable deficits in the decision‐making cognition of chronic amphetamine abusers, opiate abusers, patients with focal damage to prefrontal cortex, and tryptophan‐depleted normal volunteers: evidence for monoaminergic mechanisms. Neuropsychopharmacology. 1999;20(4):322–39. 10088133 10.1016/S0893-133X(98)00091-8

[19] Strang J, Gurling H. Computerized tomography and neuropsychological assessment in long‐term high‐dose heroin addicts. Br J Addict. 1989;84(9):1011–9. 2790264 10.1111/j.1360-0443.1989.tb00784.x

[20] Gerra G, Calbiani B, Zaimovic A, Sartori R, Ugolotti G, Ippolito L, et al. Regional cerebral blood flow and comorbid diagnosis in abstinent opioid addicts. Psychiatry Res Neuroimaging. 1998;83(2):117–26. 10.1016/s0925-4927(98)00030-4 9818737

[21] Guerra D, Solé A, Camí J, Tobeña A. Neuropsychological performance in opiate addicts after rapid detoxification. Drug Alcohol Depend. 1987;20(3):261–70. 3436258 10.1016/0376-8716(87)90036-6

[22] Lee TM, Pau CWH. Impulse control differences between abstinent heroin users and matched controls. Brain Inj. 2002;16(10):885–9. 12419001 10.1080/02699050210128915

[23] Pau CW, Lee TM, Chan SF. The impact of heroin on frontal executive functions. Arch Clin Neuropsychol. 2002;17(7):663–70. 14591849

[24] Kakko J, Svanborg KD, Kreek MJ, Heilig M. 1‐year retention and social function after buprenorphine‐assisted relapse prevention treatment for heroin dependence in Sweden: a randomised, placebo‐controlled trial. Lancet. 2003;361(9358):662–8. 12606177 10.1016/S0140-6736(03)12600-1

[25] Krook AL, Brørs O, Dahlberg J, Grouff K, Magnus P, Røysamb E, et al. A placebo‐controlled study of high dose buprenorphine in opiate dependents waiting for medication‐assisted rehabilitation in Oslo, Norway. Addiction. 2002;97(5):533–42. 12033654 10.1046/j.1360-0443.2002.00090.x

[26] First MB, Gibbon M, Spitzer RL, Benjamin LS, Williams JB. Structured clinical interview for DSM‐IV® axis ii personality disorders SCID‐II. Washington: American Psychiatric Pub; 1997.

[27] First MB, Spitzer RL, Gibbon M, Williams JB. Structured clinical interview for DSM‐IV‐TR axis I disorders, research version, patient edition. New York, NY: SCID‐I/P; 2002.

[28] Reitan RM, Wolfson D. The Halstead‐Reitan neuropsychological test battery: theory and clinical interpretation. Vol 4. Tucson, AZ: Reitan Neuropsychology; 1985.

[29] Wechsler D. WAIS‐R manual: Wechsler adult intelligence scale‐revised. New York: Psychological Corporation; 1981.

[30] Melika L. Wechsler Adult Intelligence Scale‐WAIS (Arabic edition). Cairo, Egypt: Dar EL Nahda El Arabia; 1996.

[31] Wechsler DJPC. Wechsler memory scale‐revised. 1987.

[32] IBM Corp . IBM SPSS statistics for windows, version 22.0. Armonk: IBM Corp; 2013.

[33] Prosser J, Cohen LJ, Steinfeld M, Eisenberg D, London ED, Galynker II. Neuropsychological functioning in opiate‐dependent subjects receiving and following methadone maintenance treatment. Drug Alcohol Depend. 2006;84(3):240–7. 16545923 10.1016/j.drugalcdep.2006.02.006 PMC2067988

[34] Vella‐Brincat J, Macleod AD. Adverse effects of opioids on the central nervous systems of palliative care patients. J Pain Palliat Care Pharmacother. 2007;21(1):15–25. 17430825

[35] Nestler EJ. Molecular basis of long‐term plasticity underlying addiction. Nat Rev Neurosci. 2001;2(2):119–28. 11252991 10.1038/35053570

[36] Espejo EF, Serrano MI, Caillé S, Stinus L. Behavioral expression of opiate withdrawal is altered after prefrontocortical dopamine depletion in rats: monoaminergic correlates. Neuropsychopharmacology. 2001;25(2):204–12. 11425504 10.1016/S0893-133X(01)00226-3

[37] Camí J, Farré M. Drug addiction. N Engl J Med. 2003;349(10):975–86. 12954747 10.1056/NEJMra023160

[38] McEwen BS. Stress, sex, hippocampal plasticity: relevance to psychiatric disorders. Clin Neurosci Res. 2001;1(1):19–34.

[39] Wolf O, Convit A, McHugh P, Kandil E, Thorn EL, De Santi S, et al. Cortisol differentially affects memory in young and elderly men. Behav Neurosci. 2001;115(5):1002. 11584913 10.1037//0735-7044.115.5.1002

[40] Medina KL, Shear PK, Schafer J, Armstrong TG, Dyer P. Cognitive functioning and length of abstinence in polysubstance dependent men. Arch Clin Neuropsychol. 2004;19(2):245–58. 15010089 10.1016/S0887-6177(03)00043-X

[41] Latvala A, Castaneda AE, Perälä J, Saarni SI, Aalto‐Setälä T, Lönnqvist J, et al. Cognitive functioning in substance abuse and dependence: a population‐based study of young adults. Addiction. 2009;104(9):1558–68. 19686526 10.1111/j.1360-0443.2009.02656.x

